# Abscisic acid enhances tolerance of wheat seedlings to drought and regulates transcript levels of genes encoding ascorbate-glutathione biosynthesis

**DOI:** 10.3389/fpls.2015.00458

**Published:** 2015-06-30

**Authors:** Liting Wei, Lina Wang, Yang Yang, Pengfei Wang, Tiancai Guo, Guozhang Kang

**Affiliations:** ^1^The Collaborative Innovation Center of Henan Food Crops, College of Agronomy, Henan Agricultural UniversityZhengzhou, China; ^2^The National Key Laboratory of Wheat and Maize Crop Science, College of Agronomy, Henan Agricultural UniversityZhengzhou, China; ^3^The National Engineering Research Centre for Wheat, Henan Agricultural UniversityZhengzhou, China

**Keywords:** abscisic acid, ascorbate, drought stress, glutathione, transcription level, *Triticum aestivum* L.

## Abstract

Glutathione (GSH) and ascorbate (ASA) are associated with the abscisic acid (ABA)-induced abiotic tolerance in higher plant, however, its molecular mechanism remains obscure. In this study, exogenous application (10 μM) of ABA significantly increased the tolerance of seedlings of common wheat (*Triticum aestivum* L.) suffering from 5 days of 15% polyethylene glycol (PEG)-stimulated drought stress, as demonstrated by increased shoot lengths and shoot and root dry weights, while showing decreased content of hydrogen peroxide (H_2_O_2_) and malondialdehyde (MDA). Under drought stress conditions, ABA markedly increased content of GSH and ASA in both leaves and roots of ABA-treated plants. Temporal and spatial expression patterns of eight genes encoding ASA and GSH synthesis-related enzymes were measured using quantitative real-time reverse transcription polymerase chain reaction (qPCR). The results showed that ABA temporally regulated the transcript levels of genes encoding ASA-GSH cycle enzymes. Moreover, these genes exhibited differential expression patterns between the root and leaf organs of ABA-treated wheat seedlings during drought stress. These results implied that exogenous ABA increased the levels of GSH and ASA in drought-stressed wheat seedlings in time- and organ-specific manners. Moreover, the transcriptional profiles of ASA-GSH synthesis-related enzyme genes in the leaf tissue were compared between ABA- and salicylic acid (SA)-treated wheat seedlings under PEG-stimulated drought stress, suggesting that they increased the content of ASA and GSH by differentially regulating expression levels of ASA-GSH synthesis enzyme genes. Our results increase our understanding of the molecular mechanism of ABA-induced drought tolerance in higher plants.

## Introduction

It is known that biotic and abiotic stresses (including drought) induces the generation of reactive oxygen species (ROS), such as the superoxide radical (O^·^_2_) and hydrogen peroxide (H_2_O_2_) (Liu et al., 2009; Borges et al., 2014). The accumulation of ROS damages lipids and proteins, results in cell death, and inhibits plant growth (Li et al., 2011). To alleviate ROS accumulation under stress conditions, both enzymatic and non-enzymatic antioxidants are present in plants. The enzymatic antioxidants include superoxide dismutase (SOD), catalase (CAT), glutathione reductase (GR), and ascorbate peroxidase (APX) (Asada, 1992), while the non-enzymatic antioxidants include glutathione (GSH) and ascorbate (ASA) (Li et al., 2011). The SOD catalyzes the dismutation of O^·^_2_ to O_2_ and H_2_O_2_, which is subsequently reduced to H_2_O and O_2_ by CAT, APX, GR, etc. (Asada, 1992). ASA and GSH function as cofactors of enzymes of the antioxidant pathways, both can also directly quench ROS (Hernandez et al., 2001; Hossain et al., 2012).

The plant hormone abscisic acid (ABA) regulates many important plant developmental processes and is known to induce tolerance to various abiotic stresses; e.g., drought, salt, and low temperature, suggesting that it has significant agronomic potential (Giraudat et al., 1994). The involvement of ABA in mediating drought stress has been extensively explored, and many studies have examined the mechanism of ABA action at the physiological and molecular levels (Ferrandino and Lovisolo, 2014), and their findings have already been reviewed (Verslues and Zhu, 2007; Chinnusamy et al., 2008; Mehrotra et al., 2014). Increased levels of endogenous ABA have been reported in many plant species under abiotic stress, such as drought stress (Aimar et al., 2014), and exogenous ABA decreases ROS accumulation by inducing activities or expression levels of many antioxidative enzymes, resulting in enhanced abiotic tolerance, although the data supporting this hypothesis remain inconsistent. For instance, application of exogenous ABA in drought-stressed kiwifruit plant significantly enhances the activities of guaiacol peroxidase (POD), CAT, SOD, and APX (Wang et al., 2011). In drought-stressed *Cotinus coggygia*, however, ABA decreases activities of CAT, although it also markedly increases activities of SOD and POD (Li et al., 2010).

Moreover, exogenous ABA can increase content of ASA and GSH and enhance plant tolerance to abiotic stresses (Jiang and Zhang, 2002; Liu et al., 2011). To our knowledge, the molecular mechanism of ASA and GSH biosynthesis regulated by exogenous ABA application has not been reported. Wheat is an important drought-sensitive cereal crop whose growth and grain yield are severely affected by drought stress (Doyle and Fischer, 1979; Gao et al., 2011). Polyethylene glycol (PEG) 6000 is often used to stimulate drought stress in higher plants (Xiong et al., 2010; Benesova et al., 2012). In this study, transcript levels of the genes encoding ASA-GSH cycle enzymes were measured using qPCR in ABA-treated wheat seedlings suffering from PEG-stimulated drought stress to help us further understand the molecular mechanism of ABA-enhanced drought tolerance in higher plants.

## Materials and methods

### Plant materials and growth conditions

Seeds of the common wheat (*Triticum aestivum* L.) cv. Yumai 34 were sterilized with 0.01% (m/v) HgCl_2_ followed by washing with distilled water. Sterilized seeds were grown hydroponically in full-strength Hoagland's solution (Elberse et al., 2003) in glass dishes (diameter 15 cm) in a FPG-300C-30D incubator (Ningbo Laifu Technology Co., Beijing, China) under a 14-h photoperiod, irradiance of 250 μmol m^−2^ s^−1^, day/night temperature of 25/15°C, and relative humidity of 60/75%. Each dish contained approximately 60 seedlings. After 2 weeks seedlings showed approximately three leaves, and two dishes were grown under the above conditions with fresh Hoagland medium (control, CK), two dishes were incubated with fresh Hoagland medium supplemented with PEG-6000 (15%) solution for drought treatment, and another two dishes were transferred to Hoagland solution supplemented with PEG-6000 (15%) plus 10 μM ABA (Wei et al., 2009) (PEG and ABA treatment, PEG + ABA). Solution (100 mL) was exchanged every day in all treatments. The uppermost fully expanded leaves and the longest roots of wheat seedlings were separately collected at 0, 1, 2, 3, 4, and 5 days after initiating drought stress, and were immediately frozen in liquid nitrogen and stored at −80°C before assessment of physiological parameters and gene expression profiles.

### Plant measurements and analysis

Growth parameters (plant height, root length, fresh and dry weight of root and leaf tissues) were recorded every day during the stress period. Ten individual wheat seedlings were randomly harvested from each dish. Plant heights and dry and fresh weights of roots and leaves were measured and calculated.

### Assays of malondialdehyde (MDA) and H_2_O_2_ content in leaves and roots of wheat seedlings

Lipid peroxidation was determined by estimating the MDA content using the method described by Zheng et al. (2008). The content of H_2_O_2_ was measured by monitoring the absorbance of the titanium-peroxidee complex at 390 nm, following the method of Jessup et al. (1994).

### Assays of GSH and ASA content

The content of GSH and ASA was measured according to the methods of Kampfenkel et al. (1995) and Smith (1985), respectively.

### Determination of the transcript levels of the eight genes encoding ASA–GSH cycle enzymes by qPCR

The genes encoding ASA-GSH synthesis-related enzymes are illustrated in Supplementary Figure S1. These genes are glutathione-S-transferase 1 (*GST1*), glutathione-S-transferase 2 (*GST2*), glutathione peroxidase 1 (*GPX1*), phospholipid hydroperoxide glutathione peroxidase 2 (*GPX2*), glutathione reductase (*GR*), dehydroascorbate reductase (*DHAR*), monodehydroascorbate reductase (*MDHAR*), and glutathione synthetase (*GS*) (Chen et al., 2011), and the genes encoding these enzymes were previously isolated from common wheat in our laboratory (Li et al., 2013). Total RNA was extracted using the TRIzol reagent (Invitrogen, Carlsbad, CA, USA) according to the manufacturer's instructions and treated with RNase-free DNase I (Takara Biotechnology [Dalian] Co., Ltd., Dalian, China) to remove contaminating genomic DNA. First-strand cDNAs were synthesized from 2 μg of total RNA using Super-Script II reverse transcriptase (Invitrogen, Carlsbad, CA, USA). qPCR was performed using a SYBR Premix Ex Taq (Perfect Real Time) kit (Takara Biotechnology [Dalian] Co., Ltd.) on a Light Cycler 480 Real-Time PCR System (Roche Diagnostics Ltd., West Sussex, UK) according to the manufacturer's instructions. Each reaction (20 μL) comprised 10 μL of SYBR Green Supermix (2×), 1 μL of diluted cDNA, and 0.5 μL of forward and reserve primers. The relative transcript levels were calculated using the 2^−ΔΔCt^ method, with the wheat *β-actin* (GenBank Accession no. AB181991) and glyceraldehyde 3-phosphate dehydrogenase (*GAPDH*) (GenBank Accession no. EF592180) genes as two internal controls. All primers are listed in Supplementary Table S1. Each data point was expressed as the average ± SD of three independent replicates.

### Statistical analysis

Data were analyzed statistically using one-way analysis of variance and Duncan's multiple range tests to determine significant differences among group means. Significant differences from the control values were determined at *P* < 0.05. All recorded values represent the means of the results of three replicates.

## Results

### Effects of exogenous ABA on phenotypes and growth parameters of wheat seedlings exposed to PEG-stimulated drought-stress conditions

**Figure 1:**
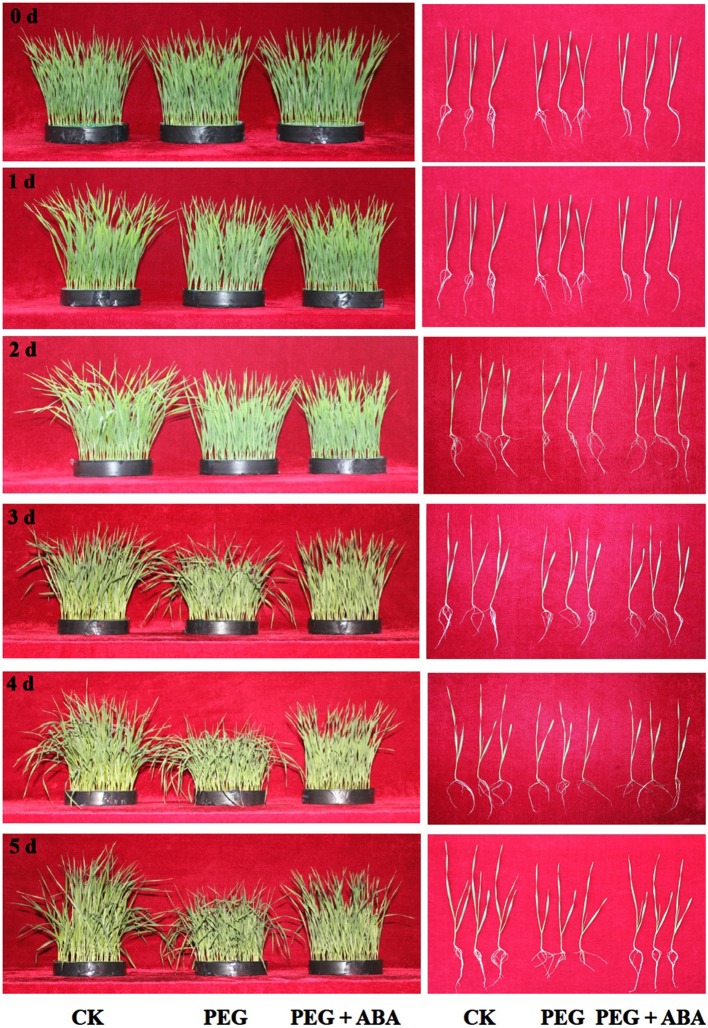
**Phenotypic changes of wheat seedlings with exogenous application of 10 μM ABA under 15% PEG-stimulated drought stress for 5 days**. CK, control; PEG, 15% PEG-6000; PEG + ABA, 15% PEG and 10 μM ABA treatment. Three independent biological replications were performed with two dishes each, and about 60 wheat seedlings were planted in a dish.

In the present study, wheat seedlings suffering from 15% PEG-stimulated drought stress showed time-dependent characteristics (Figure 1). Wheat seedlings suffering from drought stress showed no visible changes before 3 days, and then exhibited significant and deleterious phenotypes, such as curled and wilted leaves, shorter plant heights, and inhibited growth compared to control plants (Figure 1). These qualitative phenotypic effects were confirmed by quantitative analysis (Table 1). However, in the presence of exogenous 10 μM ABA, the effect of drought stress on wheat seedling growth was significantly abrogated. This was also demonstrated by the significantly increased plant heights, fresh and dry shoot weights, and fresh and dry root weights (Table 1). After 5 days, plant height, shoot fresh weight, shoot dry weight, root fresh weight, and root dry weight of PEG + ABA treated wheat seedlings were significantly higher than those of sole PEG-treated wheat seedlings by 11.4, 21.8, 21.4, 34.9, and 23.1%, respectively. These results showed that ABA improved the drought tolerance of wheat seedlings.

**Table 1:** **Effect of exogenous ABA application on growth characteristics of wheat seedlings suffering from PEG-stimulated drought stress for 5 days**.

**Growth parameters**	**Treatments**	**0 day**	**1 day**	**2 days**	**3 days**	**4 days**	**5 days**
Plant height (cm)	Control	15.06 ± 0.49	16.14 ± 0.54	17.02 ± 0.35	18.41 ± 0.49	19.35 ± 0.49^a^	22.15 ± 0.55^a^
	ABA		15.69 ± 0.55	16.34 ± 0.43	17.11 ± 0.39	17.90 ± 0.41^b^	18.98 ± 0.34^b^
	PEG		15.32 ± 0.58	15.46 ± 0.44	15.94 ± 0.35	16.16 ± 0.40^c^	16.71 ± 0.38^c^
	PEG + ABA		15.54 ± 0.56	16.01 ± 0.51	16.79 ± 0.38	17.09 ± 0.45^b^	18.61 ± 0.63^b^
Shoot fresh weight (g^·^plant^−1^)	Control	0.161 ± 0.019	0.217 ± 0.016	0.257 ± 0.012	0.262 ± 0.014	0.291 ± 0.014^a^	0.381 ± 0.014^a^
	ABA		0.190 ± 0.013	0.217 ± 0.012	0.230 ± 0.014	0.241 ± 0.011^b^	0.298 ± 0.013^b^
	PEG		0.175 ± 0.015	0.188 ± 0.018	0.194 ± 0.016	0.212 ± 0.012^b^	0.234 ± 0.017^b^
	PEG + ABA		0.184 ± 0.014	0.205 ± 0.013	0.213 ± 0.018	0.224 ± 0.013^b^	0.285 ± 0.013^b^
Shoot dry weight (g^·^plant^−1^)	Control	0.019 ± 0.002	0.022 ± 0.002	0.025 ± 0.002	0.033 ± 0.001^a^	0.037 ± 0.001^a^	0.041 ± 0.001^a^
	ABA		0.021 ± 0.001	0.023 ± 0.001	0.028 ± 0.001^b^	0.033 ± 0.001^b^	0.036 ± 0.000^b^
	PEG		0.021 ± 0.002	0.022 ± 0.000	0.024 ± 0.002^b^	0.026 ± 0.001^c^	0.028 ± 0.001^c^
	PEG + ABA		0.021 ± 0.001	0.023 ± 0.002	0.027 ± 0.001^b^	0.032 ± 0.001^b^	0.034 ± 0.001^b^
Root fresh weight (g^·^plant^−1^)	Control	0.091 ± 0.011	0.098 ± 0.014	0.124 ± 0.010	0.148 ± 0.011^a^	0.194 ± 0.010^a^	0.233 ± 0.011^a^
	ABA		0.094 ± 0.011	0.119 ± 0.011	0.129 ± 0.099^b^	0.141 ± 0.011^b^	0.157 ± 0.009^b^
	PEG		0.093 ± 0.012	0.097 ± 0.011	0.099 ± 0.010^b^	0.101 ± 0.007^c^	0.106 ± 0.009^c^
	PEG + ABA		0.092 ± 0.011	0.112 ± 0.013	0.120 ± 0.107^b^	0.130 ± 0.012^b^	0.143 ± 0.010^b^
Root dry weight (g^·^plant^−1^)	Control	0.010 ± 0.001	0.011 ± 0.002	0.015 ± 0.001	0.017 ± 0.000^a^	0.018 ± 0.000^a^	0.022 ± 0.001^a^
	ABA		0.010 ± 0.001	0.011 ± 0.001	0.014 ± 0.000^b^	0.016 ± 0.000^b^	0.017 ± 0.000^b^
	PEG		0.010 ± 0.001	0.011 ± 0.001	0.012 ± 0.000^b^	0.012 ± 0.000^c^	0.013 ± 0.000^c^
	PEG + ABA		0.010 ± 0.002	0.011 ± 0.001	0.013 ± 0.001^b^	0.015 ± 0.000^b^	0.016 ± 0.001^b^

*At 0 day of PEG-stimulated drought stress (before stress), the growth parameters of wheat seedlings are same, because PEG or ABA are not immersed in Hoagland's solution at this time point, and sizes and heights of seedlings in all treatments are uniform at this time point. Average values of 10 plants from one dish were considered as one replication and three independent biological replications were performed. Different letters indicate a significant difference at P < 0.05*.

In this study, we also found that growth of wheat seedlings was significantly inhibited by 10 μM ABA under normal conditions, which was also confirmed by qualitative and quantitative data (Figure 1, Table 1, Supplementary Figure S2). We speculated that changes in transcriptional levels in this treatment may be associated with wheat growth, and not stress tolerance. In this study, wheat seedlings treated with 10 μM ABA under normal conditions were not used in further experiments.

### MDA and H_2_O_2_ content in leaves and roots of wheat seedlings exposed to PEG-stimulated drought stress conditions in response to exogenous ABA

MDA and H_2_O_2_ content in drought and PEG + ABA treatments also increased in a time-dependent manner (Table 2). However, the MDA and H_2_O_2_ content in root and leaf tissues of PEG + ABA-treated wheat seedlings was significantly lower than those of solely PEG-treated wheat seedlings after 3 days of drought (Table 2). After 5 days of drought stress, the MDA and H_2_O_2_ content in root and leaf tissues of PEG + ABA-treated wheat seedlings were markedly lower than those of sole PEG-treated wheat seedlings by 8.7 and 31.0%, and 13.3 and 22.6%, respectively (Table 2). These results indicated that exogenous ABA application alleviated the accumulation of MDA and H_2_O_2_ induced by drought.

**Table 2:** **Effect of exogenous ABA on content of MDA and H_2_O_2_ in root and leaf tissues of wheat seedlings suffering from PEG-stimulated drought stress for 5 days**.

**Parameters**	**Treatments**	**0 day**	**1 day**	**2 days**	**3 days**	**4 days**	**5 days**
MDA content in leaf	Control	5.06 ± 0.10	4.99 ± 0.21	4.95 ± 0.17	5.00 ± 0.12	5.09 ± 0.14^c^	5.17 ± 0.14^c^
	PEG		5.25 ± 0.14	5.83 ± 0.13	6.38 ± 0.16	6.66 ± 0.13^a^	7.22 ± 0.13^a^
	PEG + ABA		5.16 ± 0.16	5.58 ± 0.12	5.91 ± 0.12	6.12 ± 0.14^b^	6.59 ± 0.14^b^
MDA content in root	Control	5.59 ± 0.31	5.55 ± 0.28	5.52 ± 0.24	5.56 ± 0.27^c^	5.45 ± 0.25^c^	5.60 ± 0.23^c^
	PEG		5.88 ± 0.35	6.40 ± 0.27	7.86 ± 0.30^a^	9.11 ± 0.28^a^	± 0.29^a^
	PEG + ABA		5.75 ± 0.28	6.08 ± 0.35	6.39 ± 0.36^b^	7.12 ± 0.32^b^	7.86 ± 0.20^b^
H_2_O_2_ content in leaf	Control	1.07 ± 0.09	1.04 ± 0.07	1.04 ± 0.05	1.08 ± 0.07^c^	1.08 ± 0.07^c^	1.02 ± 0.07^c^
	PEG		1.27 ± 0.03	1.42 ± 0.10	1.66 ± 0.09^a^	1.88 ± 0.08^a^	1.96 ± 0.11^a^
	PEG + ABA		1.18 ± 0.08	1.26 ± 0.06	1.39 ± 0.05^b^	1.55 ± 0.08^b^	1.70 ± 0.08^b^
H_2_O_2_ content in leaf	Control	1.19 ± 0.07	1.19 ± 0.01	1.19 ± 0.08	1.21 ± 0.08^c^	1.19 ± 0.10^c^	1.22 ± 0.07^c^
	PEG		1.28 ± 0.08	1.46 ± 0.05	1.75 ± 0.07^a^	2.31 ± 0.07^a^	2.57 ± 0.07^a^
	PEG + ABA		1.23 ± 0.05	1.28 ± 0.08	1.44 ± 0.05^b^	1.62 ± 0.07^b^	1.99 ± 0.06^b^

*At 0 day of PEG-stimulated drought stress (before stress), the physiological parameters of wheat seedlings are same, because PEG or ABA are not immersed in Hoagland's solution at this timepoint, and sizes and heights of seedlings in all treatments are uniform at this time point. The uppermost fully expanded leaves and longest roots of three seedlings were separately collected in one replication and three independent biological replications were performed. Different letters indicate a significant difference at P < 0.05. Units of MDA and H_2_O_2_ content, μmol.g^−1^ FW*.

### Effects of exogenous ABA on GSH and ASA content in root and leaf tissues of wheat seedlings exposed to PEG-stimulated drought stress conditions

**Figure 2:**
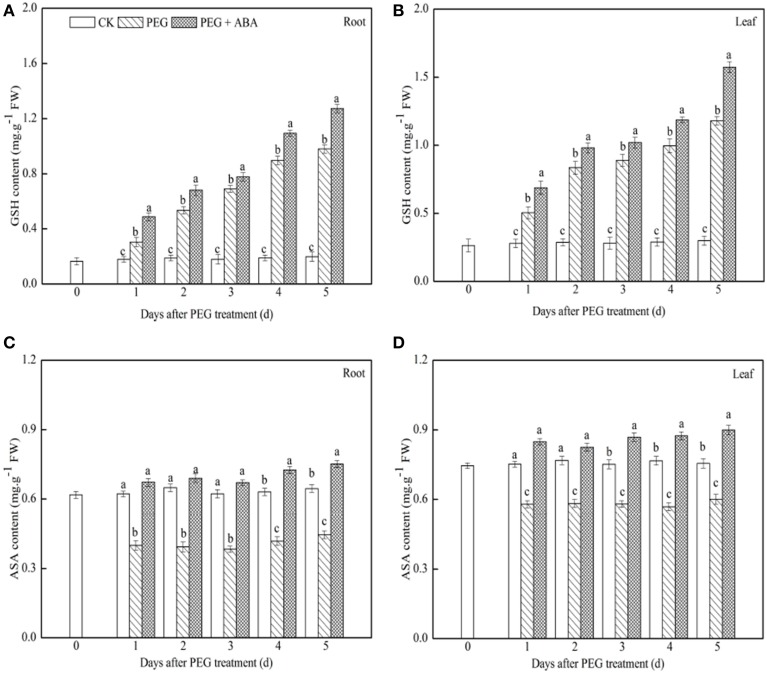
**Effects of exogenous ABA on content of GSH and ASA in roots and leaves of wheat seedlings suffered from PEG-stimulated drought stress**. **(A,C)**, GSH and ASA content in root of wheat seedling suffering from PEG-stimulated drought stress, respectively; **(B,D)**, GSH and ASA content in leaf of wheat seedling suffering from PEG-stimulated drought stress, respectively. The uppermost fully expanded leaves and longest roots of three seedlings were separately collected in one replication and three independent biological replications were performed. Different letters indicates significant differences (*P* < 0.05).

GSH content increased gradually in both leaf and root tissues of PEG-treated wheat seedlings after drought stress, and exogenous ABA application significantly accelerated this increase (Figures 2A,B). After 5 days of drought stress, GSH content in both root and leaf tissues of PEG + ABA treated wheat seedlings was 29.9 and 33.3% higher, respectively, than those in the tissues of solely PEG-treated wheat seedlings. In contrast, ASA content in root and leaf tissues of solely PEG-treated wheat seedlings decreased rapidly with prolonged drought stress, but ABA application inhibited this effect (Figures 2C,D). After 5 days of drought stress, ASA content of the root and leaf of PEG + ABA-treated wheat seedlings was 68.5 and 49.7% higher, respectively, than those in the tissues of solely PEG-treated wheat seedlings.

### Transcript levels of genes encoding enzymes involved in the ASA-GSH cycle in leaves and roots of wheat seedlings exposed to PEG-stimulated drought stress

**Figure 3:**
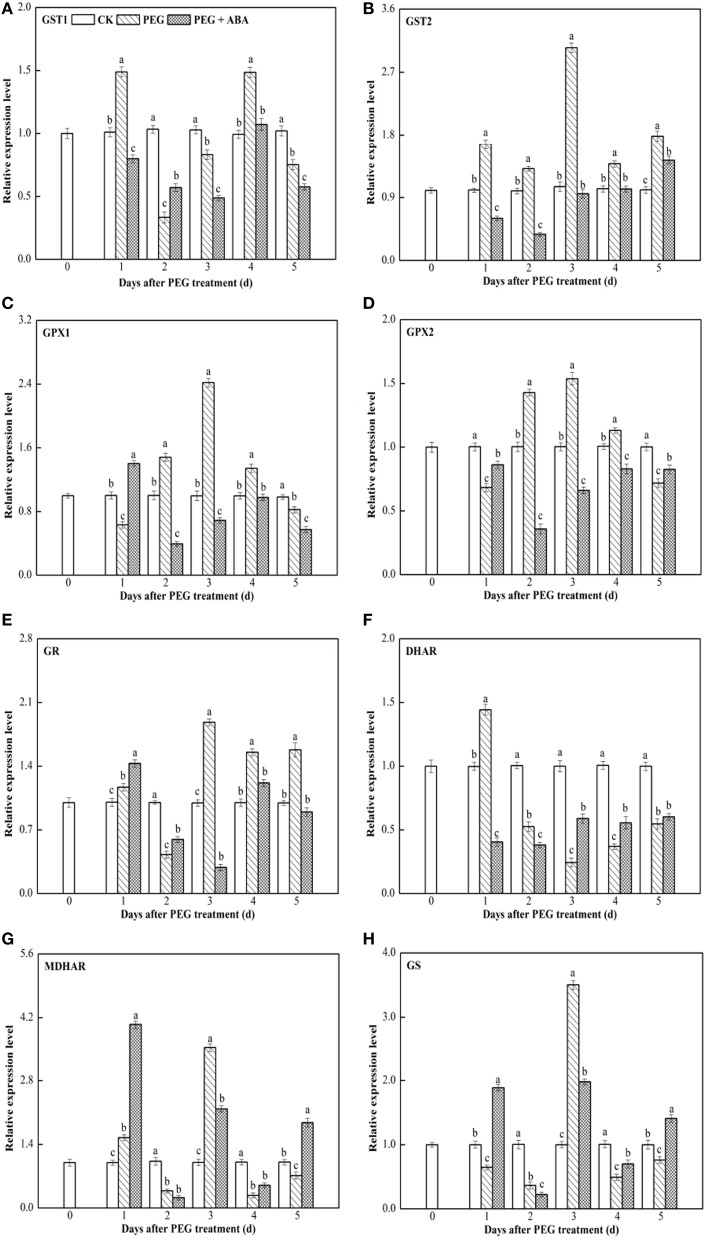
**Effects of exogenous ABA on transcript levels of the eight genes encoding ASA-GSH cycle enzymes in root of wheat seedlings suffering from PEG-stimulated drought stress**. Transcripts were analyzed by qPCR using Actin gene as internal control. **(A–H)**, transcript levels of *GST1*, *GST2*, *GPX1*, *GPX2*, *GR*, *DHAR*, *MDHAR*, and *GS* genes, respectively. The three seedlings were collected in one replication and three independent biological replications were performed. Each value is the mean ± standard deviation of three independent measurements. Different letters indicate significant differences (*P* < 0.05).

**Figure 4:**
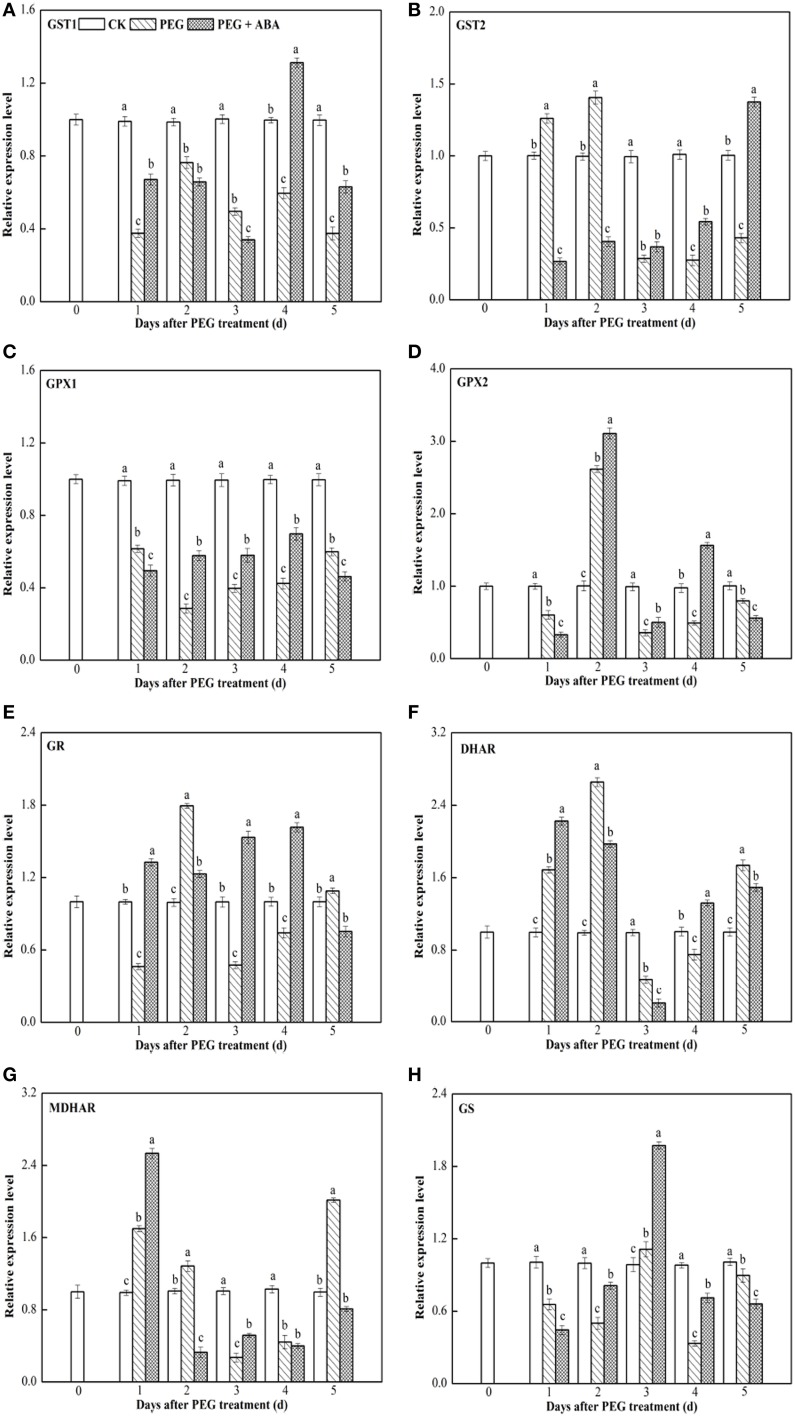
**Effects of exogenous ABA on transcript levels of the eight genes encoding ASA-GSH cycle enzymes in leaf of wheat seedlings suffering from PEG-stimulated drought stress**. Transcripts were analyzed by qPCR using *Actin* gene as internal control. **(A–H)**, transcript levels of *GST1*, *GST2*, *GPX1*, *GPX2*, *GR*, *DHAR*, *MDHAR*, and *GS* genes, respectively. The uppermost fully expanded leaves and longest roots of three seedlings were collected in one replication and three independent biological replications were performed. Each value is the mean ± standard deviation of three independent measurements. Different letters indicate significant differences (*P* < 0.05).

*GST1*, *GST2*, *GPX1*, *GPX2*, *GR*, *DHAR*, *MDHAR*, and *GS* transcript levels were measured using qPCR with the *Actin* gene as the internal control in leaf and root tissues of wheat seedlings (Figures 3, 4). Similar results were obtained using *GAPDH* gene as another internal control, as indicated in Supplementary Figures S3, S4.

In root and leaf tissues of control wheat seedlings, the expression levels of the above mentioned eight genes encoding ASA-GSH synthesis-related enzymes remained almost constant (Figures 3, 4). Under PEG-stimulated drought-stress conditions, the expression patterns of these eight genes varied in the root and leaf tissues of wheat seedlings. In roots of PEG-treated wheat seedlings, *GST1* expression was significantly induced at 1 day of stress, decreased rapidly, peaked again at 4 days, and then slowly decreased after 5 days of PEG-stimulated drought stress (Figure 3A). *GST2*, *GPX1*, *GPX2*, *GR*, *MDHAR*, and *GS* genes exhibited similar expression patterns in roots of wheat seedlings exposed to PEG-stimulated drought stress. Transcript levels of these six genes were enhanced or inhibited in root tissue within 1–2 days after initiation of PEG-stimulated drought stress, peaked at 3 days, and slowly or rapidly decreased thereafter (Figures 3B–E,G,H). *DHAR* expression in roots was significantly induced at 1 day after PEG-stimulated drought stress, whereas it decreased rapidly thereafter (Figure 3F). During the PEG-treatment period, exogenous ABA significantly increased *GST1* transcript levels at day 2 (Figure 3A), those of *GPX1* at day 1 (Figure 3C), *GPX2* at days 1 and 5 (Figure 3D), *GR* at days 1 and 2 (Figure 3E), *DHAR* at days 3, 4, and 5 (Figure 3F), and *MDHAR* and *GS* at days 1, 4, and 5 (Figures 3G,H) after PEG treatment.

During PEG-stimulated drought stress, transcript levels of eight genes encoding ASA-GSH synthesis-related enzymes in leaf differed significantly compared to those in root tissue. At all time points after PEG treatment, *GST1* and *GPX1* transcript levels were markedly inhibited (Figures 4A,C). *GST2* and *DHAR* genes were induced early, peaked at 2 days after PEG treatment, and then rapidly decreased (Figures 4B,F). *GPX2*, *GR*, and *GS* expression levels were strongly inhibited at 1 day after PEG treatment, increased and peaked separately at days 2 and 3, then increased, and rapidly decreased at subsequent time points (Figures 4D,E,H). *MDHAR* transcript levels were enhanced at 1 day after PEG treatment, whereas they decreased rapidly at subsequent time points (Figure 4G). During the PEG-treatment period, exogenous ABA significantly increased the transcript levels of *GST1* at days 1, 4, and 5 (Figure 4A), *GST2* at days 3, 4, and 5 (Figure 4B), *GPX1*, *GPX2* and *GS* at days 2, 3, and 4 (Figures 4C,D,H), *GR* at days 1, 3, and 4 (Figure 4E), *DHAR* at days 1 and 4 (Figure 4F), and *MDHAR* at days 1 and 3 (Figure 4G) after PEG treatment.

## Discussion

### Exogenous ABA enhances the tolerance of wheat seedlings suffering to PEG-stimulated drought stress

Actual soil drought stress is rarely used, because components of soil are very complicated, and it is difficult to control all soil components. In addition, it is also very difficult to discriminate water stress from other abiotic stresses in soil system. However, it is important for water stress experiment to establish a stable and controlled condition (Zhang et al., 2004). PEG have been used extensively to induce plant water deficit in a relatively controlled manner, appropriate to experimental protocols because it is a very low chronic toxicity, molecules with mol wt greater than 3000 are apparently not absorbed at all, and plant water relations can be similar whether the plants are growing in soil or in a PEG solution having an equal water potential (Kaufmann and Eckard, 1971; Mexal et al., 1975; Carpita et al., 1979).

In this study, application of exogenous 10 μM ABA decreased the growth inhibition caused by 15% PEG 6000-stimulated drought stress, as manifested by increased growth parameters (plant height, shoot and root fresh weights, and shoot and root dry weights), and decreased MDA and H_2_O_2_ content (Tables 1, 2). These suggest that exogenous ABA enhances the tolerance of wheat seedlings to drought stress, similar to the previous reports in maize, bermudagrass, and grapemvine (Todorov et al., 1998; Lu et al., 2009; Ferrandino and Lovisolo, 2014). However, ABA-enhanced drought tolerance might also be related to its protective roles, such as closing stomata, and decreasing evapotranspiration and solute uptake (Kirkham, 1983). In addition, PEG has some disadvantages in stimulating water stress, including its uptake by plants, hypoxia, and mineral contamination (Lawlor, 1970; Janes, 1974; Reid, 1978; Yaniv and Werker, 1983; Jacomini et al., 1988; Verslues et al., 1998; Blum, 2013). Thus, ABA-induced drought tolerance in wheat plant could need to be further measured under actual soil drought stress conditions.

### ABA temporally regulates the transcriptional levels of the genes encoding ASA-GSH cycle enzymes in wheat seedlings, resulting in increased GSH and ASA content

GSH and ASA are major non-enzymatic antioxidants, and the enzymes and antioxidants in the ASA-GSH cycle play important roles in scavenging of ROS (Shan and Liang, 2010; Liu et al., 2012; Rakić et al., 2014). GSH and ASA content in abiotic-tolerant plant varieties are significantly higher than those in abiotic-sensitive varieties (Vaidyanathan et al., 2003). Overexpression of the genes encoding ASA-GSH cycle enzymes in higher plants confers enhanced tolerance to abiotic stresses (e.g., salt, low temperature) by maintaining higher content of GSH and ASA (Eltayeb et al., 2006; Duan et al., 2012; Sultana et al., 2012). Our findings indicated that ASA content declined in solely PEG-treated wheat seedlings (Figure 2), indicating that PEG-stimulated drought stress could disturb synthesis of ASA. However, content of GSH increased in this treatment (Figure 2), possibly combating the oxidative stress generated due to drought stresses. This suggested that drought stress had the differential effects on between ASA and GSH synthesis. Under abiotic stresses, various expression profiles of different antioxidative enzymes and antioxidants have also been reported in previous studies (Li et al., 2010; Wang et al., 2011; Hossain et al., 2012).

In the present study, much higher content of ASA and GSH was observed in both root and leaf tissues of PEG + ABA-treated wheat seedlings (Figure 2), implying that the drought tolerance enhanced by exogenous ABA application in wheat seedlings may be related to increased content of GSH and ASA. It has been reported that there may be no post-transcriptional, translational, or post-translational regulations of the genes encoding ASA-GSH cycle enzymes (Shan and Liang, 2010; Chen et al., 2011; Liu et al., 2012). Transcriptional analysis enables quantification of changes in transcript levels of genes. Therefore, transcriptional analysis can facilitate identification of genes involved in the regulation of metabolism and provide valuable insight into the molecular mechanisms of many biosynthetic pathways (Ohdan et al., 2005). In this study, the expression levels of eight genes encoding ASA-GSH cycle enzymes were determined in PEG-stimulated drought-stressed wheat seedlings to identify their associations with the increased GSH and ASA content of wheat seedlings after exogenous ABA application.

Our results showed that, in root and leaf tissues of PEG-treated wheat seedlings to which ABA had been applied, the transcript profiles of ASA-GSH synthesis-related genes varied in a time-dependent manner, and the transcript levels of at least one of the genes were markedly enhanced by ABA application at each time point (Figures 3, 4, Supplementary Figures S3, S4). This may be associated with the increased content of GSH and ASA during the PEG-stimulated drought-stress period in these two tissues, as well as the enhanced drought tolerance of PEG + ABA-treated wheat plants (Figure 1). These results are also supported by several previous studies of maize and wheat, in which the transcript levels of diverse genes encoding ASA-GSH cycle enzymes were temporally regulated by SA under cold- and salt-stress conditions (Liu et al., 2012; Li et al., 2013).

### Transcriptional patterns of ASA-GSH synthesis-related genes in root and leaf tissues of PEG-treated wheat seedlings treated with exogenous ABA

Figures 3, 4 and Supplementary Figures S3, S4 show that the root and leaf tissues of drought-stressed wheat seedlings exhibited similar transcriptional patterns of ASA-GSH synthesis genes after pretreatment with ABA. Transcript levels of several genes at various time points, such as *GR* at day 1, *DHAR* at day 4, *MDHAR* at day 1, and *GS* at day 4, were increased by ABA in both the roots and leaves of PEG-treated wheat seedlings (Supplementary Table S2). These results suggested that root and leaf tissues may show similar responses to exogenous ABA application. However, differences in the transcriptional patterns in root and leaf tissues were also observed. For example, transcript levels of *GST1* at days 1 and 4, *GST2* at days 3, 4, and 5, *GPX1* at days 2, 3, 4, *GR* at days 3 and 4, and *GS* at days 2 and 3 significantly increased in the leaves of wheat seedlings subjected to PEG + ABA treatment, whereas transcript levels of these genes markedly decreased at the above time points in root tissue (Supplementary Table S2). These suggest that the mechanisms of action of ABA differ between root and leaf tissues of wheat seedlings. The differences in the transcript levels of ASA-GSH synthesis-related genes identified between root and leaf tissues may be related to differences in GSH and ASA content in the two tissues (Figure 2), or to the different functions, growth environments, and sensitivities of roots and leaves to PEG-stimulated drought stress and ABA. The difference in the transcriptional profiles of root and leaf tissues further suggests that exogenous ABA application may have profound and distinct effects on these two tissues.

### Effects of ABA and other plant hormones on the transcriptional profiles of genes encoding ASA-GSH synthesis-related enzymes under PEG-stimulated drought stress

qPCR has been used previously to examine the transcriptional profiles of genes encoding ASA-GSH synthesis-related enzymes regulated by SA in drought- and salt-stressed wheat seedlings, and cold-stressed eggplant (Chen et al., 2011; Kang et al., 2013; Li et al., 2013). The transcriptional profiles of genes encoding ASA-GSH synthesis-related enzymes in the leaf tissue of PEG-treated wheat seedlings in the present study were compared to our previous findings on genes regulated by SA (Kang et al., 2013), because there are many similarities between the two studies; e.g., use of identical materials (leaf tissue of wheat seedlings), experimental conditions (15% PEG-stimulated drought stress), and several sampling time-points (1 and 3 days after initiation of PEG-stimulated drought stress). Expression of *GST* at day 1, *GST2* at day 3, *GPX1* at day 2, *GR* at days 1 and 3, *DHAR* at days 2 and 3, *MDHAR* at day 1, and *GS* at day 2 were induced by both ABA and SA in the leaf tissue of wheat seedlings under PEG-stimulated drought stress (Supplementary Table S3). However, the expression profiles of the majority of genes encoding ASA-GSH synthesis enzymes were differentially regulated by ABA and SA under PEG-stimulated drought conditions (Supplementary Table S3). These results suggest that multiple plant hormones may increase the GSH and ASA content, possibly by differentially regulating the expression of genes encoding ASA-GSH synthesis enzymes.

## Conclusions

Exogenous application of 10 μM ABA significantly enhanced the tolerance of wheat seedlings to PEG-stimulated drought stress, as shown by alleviated growth inhibition, reduced content of MDA and H_2_O_2_, and increased content of GSH and ASA in the root and leaf tissues. The increased GSH and ASA content may be associated with upregulated expression levels of ASA-GSH synthesis enzyme genes in time- and organ-specific manners. The transcriptional profiles of eight genes encoding ASA-GSH synthesis enzymes regulated by ABA differed between the root and leaf tissues of PEG-treated wheat seedlings. Comparison of our results with the findings of previous studies showed that, under PEG-stimulated drought-stress conditions, ABA and SA induced differential transcriptional profiles of genes encoding ASA-GSH synthesis enzymes in wheat seedlings. Our findings can provide specific information on the molecular mechanisms of the ASA and GSH synthesis regulated by ABA in drought-stressed plants.

## Author contributions

Liting Wei and Lina Wang performed the main experimental work, Liting Wei wrote the manuscript, Yang Yang and Pengfei Wang were responsible for viability tests and statistical analysis, Tiancai Guo designed the experiments, and Guozhang Kang provided the financial support and revised the manuscript.

### Conflict of interest statement

The authors declare that the research was conducted in the absence of any commercial or financial relationships that could be construed as a potential conflict of interest.

## Abbreviations

ABA: abscisic acid
ASA: ascorbate
DHAR: dehydroascorbate reductase
GPX: glutathione peroxidase
GR: glutathione reductase
GS: glutathione synthetase
GSH: glutathione
GST: glutathione-S-transferase
H_2_O_2_: hydrogen peroxide
MDA: malondialdehyde
MDHAR: monodehydroascorbate reductase
PEG: polyethylene-glycol
SA: salicylic acid.
