# Supplementary material for: Abscisic acid enhances tolerance of wheat seedlings to drought and regulates transcript levels of genes encoding ascorbate-glutathione biosynthesis
Source: Front Plant Sci. 2015 Jun 30;6:458. doi: 10.3389/fpls.2015.00458 (PMC4485351; doi:10.3389/fpls.2015.00458)
Supplement: Supplementary file 1 [file Table1.DOC]

**Supplemental Table 1. DNA sequences of PCR primers were used for qPCR determination of the eight ASA-GSH biosynthesis-related genes in wheat seedlings.**

| Genes | Accession no. | Primer pairs | Expected amplification sizes (bp) |
| --- | --- | --- | --- |
| *GST1* | JX051003 | F: GACGAGGCGTGGAAGGACGGCT | 113 |
|  |  | R: GGGAAGATGGCGGCGTTGC |  |
| *GST2* | JX051004 | F: AGCTCTTGGCGTCTTGGCT | 131 |
|  |  | R: AGGCTTCCCCTTGGAGCAC |  |
| *GPX1* | AF475124 | F: CTCGCTTCAAGGCTGAGTA | 97 |
|  |  | R: CCACCTTTGCTAGACTTCAG |  |
| *GPX2* | JN578723 | F: CCTAACTAACTCCAACTACACC | 105 |
|  |  | R: TCCTGCCCACCAAACTGAT |  |
| *GR* | AY364467 | F: ATGAATACTCCCGTACATCAGT | 55 |
|  |  | R: TTTGTTACATCACCCACAGC |  |
| *DHAR* | AY074784 | F: GTGCCTGTGTATAACGGTG | 94 |
|  |  | R: ACAAGTGATGGAGTTGGGT |  |
| *MDHAR* | AK371371 | F: AGAAGTTTACGCCCTTCGGC | 132 |
|  |  | R: TTGGAATGTCATCGCCATC |  |
| *GS* | AJ579382 | F: ATCGCCAAGCTCCGTCAATG | 88 |
|  |  | R: ACAAGTCAGGGTTTTCAATCG |  |
| *Actin* | AB181991 | F: AGCGGTCGAACAACTGGTA | 101 |
|  |  | R: AAACGAAGGATAGCATGAGGAAGC |  |
| *GAPDH* | EF592180 | F: TTTTCACCGACAAGGACA | 179 |
|  |  | R:AAGAGGAGCAAGGCAGTT |  |

Notes: F, forward primer, R, reverse primer.
