# Supplementary material for: Abscisic acid enhances tolerance of wheat seedlings to drought and regulates transcript levels of genes encoding ascorbate-glutathione biosynthesis
Source: Front Plant Sci. 2015 Jun 30;6:458. doi: 10.3389/fpls.2015.00458 (PMC4485351; doi:10.3389/fpls.2015.00458)
Supplement: Supplementary file 2 [file Table2.DOC]

**Supplemental Table 2. Comparisons on the transcription levels of the genes encoding ASA-GSH cycle enzymes between the root and leaf of ABA-treated wheat seedlings suffered from PEG-stimulated drought stress.**

| Expression profiles of the genes encoding ASA-GSH cycle enzymes between the root and leaf tissues | Gene names and expression patterns |
| --- | --- |
| Commonly upregulated by ABA in both root and leaf of PEG-treated wheat seedlings | *GR* at 1 day, *DHAR* at 4 day, *MDHAR* at 1 day, *GS* 4 at day |
| Specially upregulated by ABA in the root of PEG-treated wheat seedlings | *GST1* at 2 day, *GPX*1 at 1 day, *GPX2* at 1 and 5 days, *GR* at 2 day, *DHAR* at 3 and 5 days, *MDHAR* at 4 and 5 days, *GS* at 1 and 5 days |
| Specially upregulated by ABA in the leaf of PEG-treated wheat seedlings | *GST1* at 1 and 4 days, *GST2* at 3, 4 and 5 days, *GPX1* at 2, 3 and 4 days, *GPX2* at 2, 3 and 4 days, *GR* at 3 and 4 days, *DHAR* at 1 day, *MDHAR* at 3 day, *GS* at 2 and 3 days |
