# Supplementary material for: Abscisic acid enhances tolerance of wheat seedlings to drought and regulates transcript levels of genes encoding ascorbate-glutathione biosynthesis
Source: Front Plant Sci. 2015 Jun 30;6:458. doi: 10.3389/fpls.2015.00458 (PMC4485351; doi:10.3389/fpls.2015.00458)
Supplement: Supplementary file 3 [file Table3.DOC]

**Supplemental Table 3. Comparisons on the transcription levels of the genes encoding ASA-GSH cycle enzymes between ABA- and SA-treated wheat seedlings suffered from 15% PEG-stimulated drought stress.**

| Expression profiles of the genes encoding ASA-GSH cycle enzymes regulated by ABA and SA hormones | Gene names and expression patterns |
| --- | --- |
| Commonly upregulated by ABA and SA in the leaf of PEG-treated wheat seedlings | *GST1* at 1 day, *GST2* at 3 day, *GPX1* at 2 day, *GR* at 1 and 3 days, *MDHAR* at 1 day, *GS* at 2 day |
| Specially upregulated by ABA in the leaf of PEG-treated wheat seedlings | *GST1* at 1 day, *GPX1* at 3 day, *GPX2* at 2 and 3 days, *DHAR* at 1 day, *MDHAR* at 3 day, *GS* at 3 day |
| Specially upregulated by SA in the leaf of PEG-treated wheat seedlings | *GST1* at 2 and 3 days, *GST2* at 1 and 2 days, *GPX1* at 1 day, *GPX2* at 1 day, *GR* at 2 day, *MDHAR* at 2 day, *GS* at 1 day |
