# Supplementary material for: Abscisic acid enhances tolerance of wheat seedlings to drought and regulates transcript levels of genes encoding ascorbate-glutathione biosynthesis
Source: Front Plant Sci. 2015 Jun 30;6:458. doi: 10.3389/fpls.2015.00458 (PMC4485351; doi:10.3389/fpls.2015.00458)
Supplement: Supplementary file 4 [file DataSheet1.PDF]

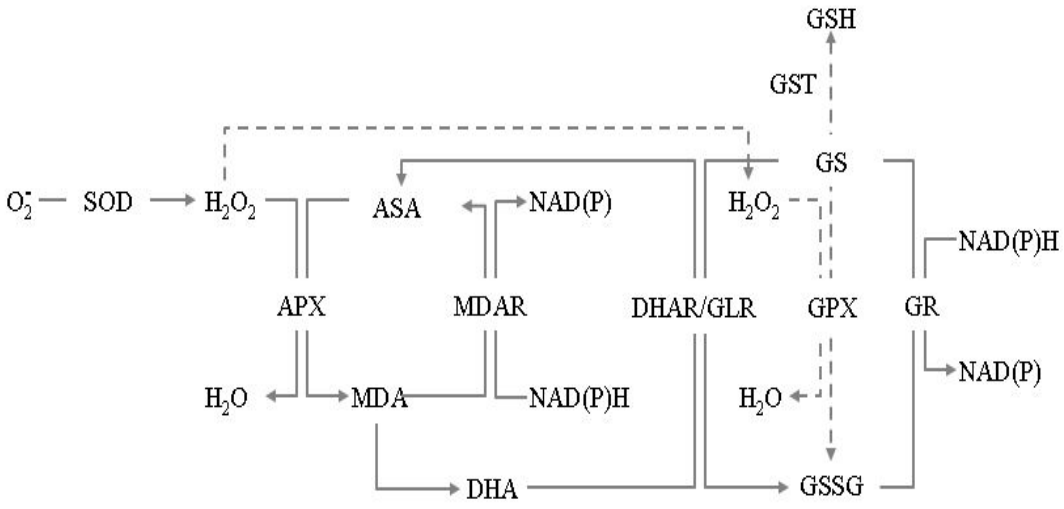

Supplementary Fig. S1 Putative metabolic interaction of ASA- and GSH-based antioxidant system modified from Hossain et al., (2012) .
