# Supplementary material for: Abscisic acid enhances tolerance of wheat seedlings to drought and regulates transcript levels of genes encoding ascorbate-glutathione biosynthesis
Source: Front Plant Sci. 2015 Jun 30;6:458. doi: 10.3389/fpls.2015.00458 (PMC4485351; doi:10.3389/fpls.2015.00458)
Supplement: Supplementary file 5 [file DataSheet2.PDF]

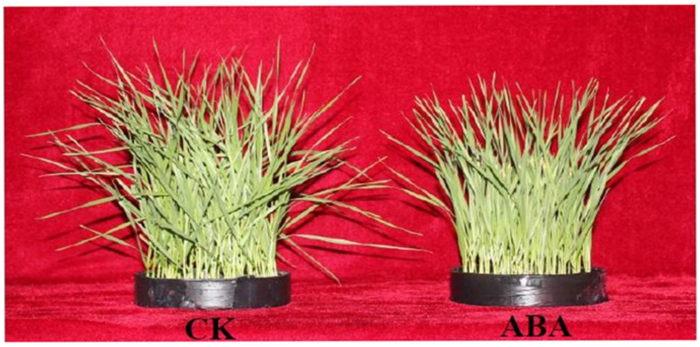

Supplemental Fig. S2 Phenotypic changes of wheat seedlings treated with exogenous application of 10 μM ABA for 5 days.
