# Supplementary material for: Abscisic acid enhances tolerance of wheat seedlings to drought and regulates transcript levels of genes encoding ascorbate-glutathione biosynthesis
Source: Front Plant Sci. 2015 Jun 30;6:458. doi: 10.3389/fpls.2015.00458 (PMC4485351; doi:10.3389/fpls.2015.00458)
Supplement: Supplementary file 7 [file DataSheet4.PDF]

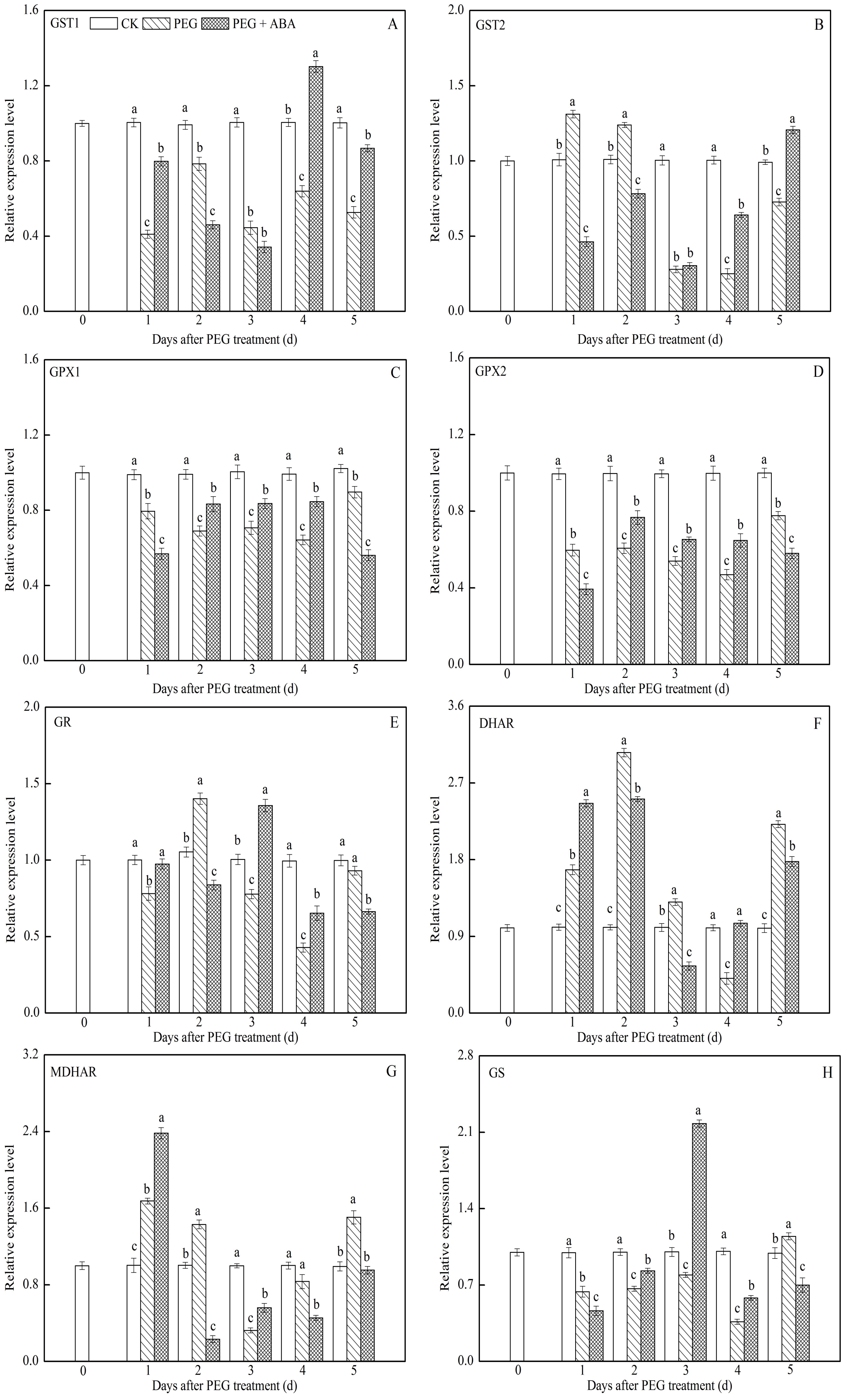

GST1
CK
PEG
PEG + ABA
A
GST2
B
GPX1
C
GPX2
D
GR
E
DHAR
F
MDHAR
G
GS
H
Relative expression level
Days after PEG treatment (d)
0
1
2
3
4
5

Supplemental Fig. S4. Effects of exogenous ABA on the transcript levels of the eight genes encoding ASA-GSH cycle enzymes in leaf of wheat seedling suffered from PEG-stimulated stress. Transcripts were analyzed by qPCR using *GAPDH* gene as internal control. (A-H), transcript levels of *GST1*, *GST2*, *GPX1*, *GPX2*, *GR*, *DHAR*, *MDHAR*, and *GS* genes, respectively. Each value is the mean ± standard deviation of at least three independent measurements. Different letters indicate significant differences ($P<0.05$).
